# Development and content validity of the Lupus Foundation of America rapid evaluation of activity in lupus (LFA-REAL™): a patient-reported outcome measure for lupus disease activity

**DOI:** 10.1186/s12955-019-1151-8

**Published:** 2019-06-07

**Authors:** Anca D. Askanase, R. Paola Daly, Miya Okado, Kayla Neville, Avery Pong, Leslie M. Hanrahan, Joan T. Merrill

**Affiliations:** 1 Columbia University Medical Center, 630 West 168th Street, P&S 3-3450, New York, NY 10032 USA; 2 Lupus Foundation of America, 2121 K Street, Suite 200, Washington, DC 20037 USA; 3 Department of Clinical Pharmacology, Oklahoma Medical Research Foundation, 825 NE 13th Street, Oklahoma City, OK 73104 USA

**Keywords:** Lupus, SLE, Disease activity, Measure, Experience, PRO

## Abstract

**Background/purpose:**

The LFA REAL™ is a measurement system for evaluating lupus disease activity from both clinician and patient perspectives. Patients’ viewpoints are captured using a patient-reported outcome (PRO) questionnaire. A series of visual analog scales are designed to rate disease severity and progress over the past 4 weeks. Brief instructions guide the patient to distinguish between active, potentially reversible symptoms and chronic pain or discomfort that are more likely due to damage. Beyond its simplicity and efficiency, the PRO can provide versatile assessments from a global, organ-based, and symptom-specific level. This paper describes the patient-centered approach used to evaluate the content validity of the LFA-REAL PRO.

**Methods:**

The PRO was developed in accordance with FDA guidance. A two-phase qualitative study was performed with 25 lupus patients, 10 who participated in concept elicitation (Phase 1) and 15 in cognitive debriefing interviews (Phase 2). Qualitative data were analyzed using ATLAS.ti software v7.5. Upon completion of the interviews, participants completed the draft PRO and additional measures to characterize the sample.

**Results:**

The mean age of participants was 45.6 and 88% were female, as expected in a lupus population. The mean SF-36 physical component score was 29.8 and the mean mental component score was 46.4. Phase 1 elicited symptom saturation and mapping of the draft PRO. Fatigue was reported by 100% of patients, highlighting its importance as a measurable domain. Additionally, 100% of patients spontaneously mentioned arthritis, which may be more important to this group than previously estimated, substantiating the approach of this PRO to break down components of arthritis into joint pain, stiffness, and swelling. Shortness of breath and fever were reported more frequently than expected. Phase 2 data demonstrated that participants found the instrument easy to use and offered recommendations to improve clarity, leading to adjustments in wording and formatting.

**Conclusions:**

Results suggest that the LFA-REAL PRO has content validity and, with some modifications suggested by participants, is ready for quantitative validation, including tests of reliability, validity, responsiveness to change, and performance relative to other PROs used in lupus trials. After validation, the LFA-REAL system is intended for use in clinical practice and research.

**Electronic supplementary material:**

The online version of this article (10.1186/s12955-019-1151-8) contains supplementary material, which is available to authorized users.

## Background

Lupus is a heterogeneous autoimmune disease characterized by periods of flares and remission, with a high impact on quality of life. Patients with increased lupus disease activity are known to have poor quality of life [1]. Disease activity is traditionally assessed by physicians on the basis of non-systematic history taking, physical examinations, and interpretation of laboratory test results, which may not be available for weeks. Validated, clinician-derived outcome measures that are commonly used in clinical trials include the British Isles Lupus Activity Group (BILAG), SLE Disease Activity Index (SLEDAI), Physician’s Global Assessment (PGA), and composite indices based on these instruments, none of which are simple or easy to assess in a busy clinic [2, 3]. Patient-reported outcomes (PRO) are usually included in trial results, but rarely as a key endpoint and are not usually used in clinical practice in the care of SLE patients, despite the fact that their pivotal importance in good medical care has been increasingly recognized [4, 5].

The difficulty in using PRO as measures of disease activity in lupus clinical trials stems from the discordance between the physician and patient estimates of disease activity [6–11]. Potential reasons behind this discordance have been studies by some and point to the relative importance paid to different concepts by physicians vs. patients in evaluating disease activity [12, 13]. Although this may be due to physician misjudgment of the degree of change experienced by patients, the discrepancy between the clinician and patient reports might also suggest that patients incorporate drug side effects or other co-morbidities in with lupus activity while physicians are trained to consider those as separate entities. While knowing whether to attribute symptoms to active lupus disease is often challenging for patients and their clinicians, instructing patients to think about this difference when scoring the PRO should improve the likelihood that disease activity is being scored, as opposed to permanent damage or side effects of medications. Neglect of patient input could affect the accuracy of trial results, the quality of patient care decisions, as well as the patients’ adherence to prescription medication, compliance with follow-up appointments, and trust in their care. The parallel assessment of the most common symptoms using the PRO/ClinRO might help explain the frequent discordance between patient and physician evaluations.

The recent publication by Holloway reviewed in detail al the PROs in the lupus arena. There are more than 60 PRO measures that have been used in studies of lupus, with only a few that are SLE-specific, and fewer still that have provided guidance to try and distinguish active lupus symptoms from treatment side effects or the collateral damage of a chronic disease [14]. No existing PRO measure integrates correlative assessments between patients and their doctors to facilitate working together to evaluate disease activity and making fully informed treatment decisions. The Systemic Lupus Activity Questionnaire (SLAQ) is a validated disease activity PRO, derived from the SLAM, that has been validated and used in epidemiological research [15]. Symptom checklists have been proposed by others [16]. Additionally, the SIMPLE Index that includes patient reported items, medication use and laboratory tests was developed as a surrogate for disease activity [17]**.** The Lupus Foundation of America—Rapid Evaluation of Activity in Lupus (LFA-REAL) system includes both clinician-reported outcomes and patient-reported ratings developed in parallel that capture complementary information. Studying and validating these instruments together may create a more complete picture of lupus disease activity, while helping to distinguish between discordance based on differing opinions and discordance based on addressing completely different topics. The LFA-REAL PRO is different from the SLAQ and symptom checklists by focusing on continuous quantification of current symptoms, with specific instructions aimed at distinguishing active disease from damage and depression.

The main advantages of a disease activity-driven PRO that correlates with a ClinRO are: 1. By including a focus on overlapping assessment approaches, this could be used in practice as a way to better communicate about symptom progress. In a patient centered care model this might improve the relationship between providers and patients by improving communication, trust, and adherence to treatment plans. 2. Could be self-administered and used to alert the health care providers of a change in health status that would prompt an earlier in-person evaluation of a patient. 3. Could be used in clinical trials as outcome measures. The inclusion in the PRO of both patient-based and clinician-based assessment approaches, differences between the assessments might be better understood as either a different focus or a different assessment with the same focus. 4. By improving the correlation between clinician and patient-reported outcomes, both could be used by practitioners to justify need for increased therapy and use of biologics by payers. 5. Could be used in clinical epidemiological research to provide a better proxy for disease activity measures that cannot be obtained without in-person visits. 6. Could allow for increased uptake in the community based on the possibility to decrease the duration of assessments while increasing meaningful communications. The providers and the patients would more effectively and precisely interact, resulting in shorter visits. 7. Could be scaled in prospective studies to provide a clinically meaningful target for therapy for a treat-to-target approach in everyday care.

The FDA Clinical Outcomes Assessment PRO guidance recommends using an iterative process of qualitative research involving concept elicitation to identify the concepts that are most important to patients and cognitive debriefing interviews (to ensure that the draft PRO is easy to understand and use by patients), in order to assure content validity of the measure in the target population [18–22]. Steps to ascertain content validity of the PRO were important to address before the two instruments could be studied side by side. The purpose of this paper is to describe the methods and results of the qualitative work performed to complete this process.

Early work that included focus groups, patient and stakeholder input informed the development of a PRO to match the CLIN-RO. The health domains of the PRO were tested prior to the work described in this paper [23]. Further concept identification included six focus groups (*N* = 35 patients) conducted by the LFA staff in different geographical locations (New York, Atlanta, Oklahoma, Boston, Washington DC, Los Angles) and was analyzed in a full report [24].

## Methods

This qualitative study was performed through interviews with 25 adult participants with lupus from the Washington D.C. area. The interviews were conducted by Evidera, a healthcare research firm with expertise in outcomes research, instrument development, and validation. The first phase included 10 patients in 45–60 min concept elicitation interviews conducted by telephone. The second phase included 15 patients with in-person or over the phone cognitive debriefing interviews.

Prior to the interviews, IRB approval was obtained. Written informed consent and a medical release of information form were obtained from the participants. Interviews were performed by experienced qualitative interviewers. A semi-structured interview guide for each phase was used to ensure consistency across interviews. In addition to the questions asked during the interviews, participants were invited to complete the LFA-REAL PRO questionnaire, a health-related quality of life questionnaire, and a socio-demographic form. All interviews were audio-recorded. Participants’ treating physicians were contacted to complete a brief clinical form to confirm lupus diagnosis.

### Recruitment of participants

The patient registries and networks of the Lupus Foundation of America (LFA) were used to identify potential participants. Efforts were made to recruit a diverse sample representative of the lupus population, including men, women, and people of African American and Hispanic origin. Interested respondents were directed to the LFA website for more information and to complete an online interest form containing pre-screening questions. If the person seemed likely to meet the eligibility requirements (see below) based on the online interest form, a member of the LFA staff conducted a full screening over the phone using a standardized recruitment script to verify the information provided in the survey. LFA staff tracked recruitment efforts using a secure, password-protected participant recruitment tracking log. Enrolled participants were scheduled for interviews either by telephone, on-site at Evidera, or at the LFA office.

Participant flow chart is included in the Additional file 1 section. A total of 25 participants were recruited to participate in the study (Phase I and Phase II). Out of the 665 that responded to the online screening questions, 435 completed the online screening form successfully and of those, 219 were found to be ineligible. The most common reason for ineligibility was the participant’s willingness to participate in a telephone or in-person interview. Among the 216 who were found to be eligible based on the online pre-screening questions, we were unable to reach 33 and did not need to contact 153, as additional participants were not needed once content saturation was achieved. The remaining 30 were eligible and immediately enrolled in the study. Only 26 of the 30 were successfully scheduled, with 25 fully completing their interview. The remaining participant did not show up for the interview. Of the 25 people interviewed in the study, ten participated in phase I concept elicitation telephone interviews and 15 participated in phase II cognitive interviews (*n* = 11 by telephone, *n* = 2 in-person at the LFA office in Washington D.C. and n = 2 in-person at the Evidera office in Bethesda, MD).

Participants who met the following inclusion/exclusion criteria were invited to participate in the study.

**Inclusion Criteria:** ≥18 years of age at the time of consent; medical diagnosis of SLE; able to read, speak, and understand English sufficiently to complete all assessments; willing and able to provide written informed consent to participate in research; and willing and able to provide a signed medical release form to verify SLE diagnosis with the treating physician.

**Exclusion Criteria:** the presence of a cognitive impairment, hearing difficulty, visual impairment, acute psychopathology, or insufficient knowledge of the interview language that, in the opinion of the investigator/interviewer, could interfere with his or her ability to provide written consent and complete an interview; previous participation in any study related to the development of the LFA-REAL PRO; currently participating in an interventional research study; unable to complete the study procedures; or unwilling to have the interview audio-recorded.

#### Phase I (concept elicitation)

Participants were asked questions about their experience with lupus, including symptoms, treatment, and how lupus impacts their daily life. Participants were mailed the forms which they completed and returned the forms to Evidera using a pre-paid mailing envelope.

The primary objective of the concept elicitation interviews was to elicit emergent descriptions of the participants’ experience of lupus. These included concepts related to kinds of lupus symptoms, information regarding disease activity and worsening of symptoms, the impact of lupus features on daily function and quality of life, and other aspects of the lupus condition that patients consider important. Participants were also asked to provide feedback on the draft LFA-REAL PRO questionnaire that they completed during the interview.

#### Phase II (cognitive debriefing interviews)

Cognitive debriefing interviews focused on eliciting feedback regarding the following items: 1) clarity of each item; 2) interpretability of each items 3) ease of completion of the instrument; 4) comprehensiveness of the instrument; and 5) appropriateness of the format, instructions, response scales, and recall period used.

A semi-structured interview guide was used to guide the interview. Participants were asked to complete the LFA-REAL PRO and to provide feedback on its content, structure, response options, and understandability. At the completion of the interview, participants completed the SF-36 questionnaire and the socio-demographic and clinical questionnaire. All interviews were audio-recorded.

#### Semi-structured interview guide

Standardized semi-structured interview guides were used in the concept elicitation and cognitive debriefing interview phases. The semi-structured interview guide for Phase I was developed based on the results of preliminary concept identification work conducted by the LFA. The semi-structured interview guide for Phase II was developed based on the results from Phase I and the content of the LFA-REAL PRO.

### Measures

#### LFA-rapid evaluation of activity in lupus PRO (LFA-REAL PRO)

**Fig. 1:**
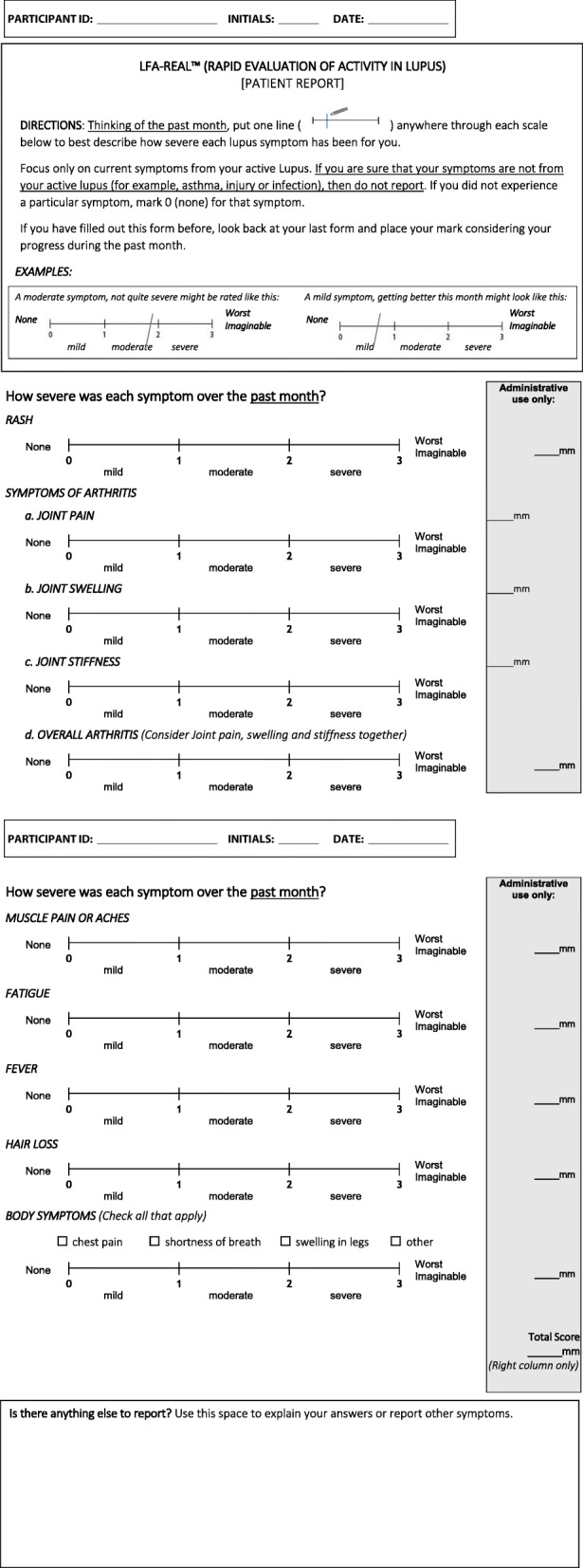
LFA-REAL™ (RAPID EVALUATION OF ACTIVITY IN LUPUS) [PATIENT REPORT]

With a recall period of four weeks, the LFA-REAL PRO asks patients to evaluate the severity and progress of symptoms associated with their current lupus activity along a series of anchored visual analog scales (VAS). Items evaluated separately include rash, symptoms of arthritis (i.e., joint pain, joint swelling, and joint stiffness), a summary arthritis score that integrates those three features, muscle pains or aches, body symptoms (e.g., shortness of breath), fatigue, fever, and hair loss. These items evolved and were modified in Phase II based on patient concept elicitation results, and the final instrument incorporated feedback from Phase II. A pre-defined component of the instrument was to break arthritis and mucocutaneous symptoms down into discrete components that might allow patients to describe similar features to those that physicians consider in the LFA REAL™ CLINRO. For example, a score for overall arthritis is not elicited until after the patient scores each component of joint pain, joint stiffness, and joint swelling, in effect, teaching patients through this process how physicians evaluate arthritis. The LFA-REAL was not shown to the patients until after the concept elicitation and cognitive debriefing interviews were completed, both of which confirmed the validity of the descriptors and provided edits to the wording used for the instructions and labels. The current version of the PRO is presented in Fig. 1.

#### SF-36 (version 1.0 standard; Rand scoring) questionnaire

This 36-item questionnaire reports current experiences or experiences over the past four weeks using Likert-type response scales to assess patient health status and quality of life. Scores range from 0 to 100, with higher scores indicating higher levels of functioning. Scores were generated for each of the eight domains (i.e., physical functioning, role limitations due to physical problems, bodily pain, general health perceptions, vitality, social functioning, and role limitations due to emotional problems and mental health) and two summary domains (i.e., physical component summary [PCS] and mental component summary [MCS]).

The SF-36 was chosen to describe the population based on its extensive use in SLE studies [25]. We acknowledge the shortcomings of the SF-36 in SLE and its potential for floor effects and in the validation study we plan to include several other PROs [26]. SF-36 scoring was based on the original orthogonal rotation [27, 28].

#### “Questions about You” form (a socio-demographic and clinical questionnaire)

Participants completed the brief “Questions About You” form, a socio-demographic and clinical questionnaire, after completing the semi-structured interviews. The “Questions About You” form asked participants to provide socio-demographic information including, but not limited to, age, race, employment, education, and basic clinical information. This information was used for descriptive purposes to characterize the population sample.

#### Clinical form

For eligible participants, treating physicians were asked to complete a clinical form to confirm and date the diagnosis of lupus.

### Data analysis

#### Quantitative data

A database for all quantitative data collected during the study was developed, tested, and validated using DataFax system, a direct fax-to-computer software using optical character recognition (OCR) to collect study case report forms (CRFs). DataFax is an FDA Title 21 Code of Federal Regulations Part 11 Compliant system that provides a time-stamped electronic audit trail for the creation, modification, or deletion of electronic data.

Electronic images of the faxed CRFs (sociodemographic, clinical data and questionnaire item scores) were entered into the database and reviewed by project scientific staff. Data discrepancies were identified and resolved.

#### Qualitative data

All interviews were digitally recorded, and audio recordings were transcribed. An Evidera project team member reviewed the interview transcripts for content and removed any participant-identifying information. The de-identified transcript was used for analysis. Qualitative data was analyzed using ATLAS.ti qualitative data analysis software version 7.5, which is designed for the qualitative analysis of textual, graphical, audio, and video data. It is a concept database that allows the researcher to create and enter names of concepts, or “codes,” to be used for conceptualizing large amounts of qualitative data. The program allows the analyst to organize and relate these concepts to each other in order to evaluate the underlying structure of the qualitative data.

A coding dictionary was developed by Evidera based on the structure of the interview guide and other concepts of interest, and imported to ATLAS.ti. Two different staff members independently reviewed and coded the first interview transcript, and the results were compared and reconciled. Once there was sufficient agreement between the initial coders, the rest of the transcripts were processed. Concepts mentioned within each transcript were assigned codes from the coding dictionary. If new codes were identified, they were added to the coding dictionary. Outputs were generated based on these codes, which were entered into a saturation grid in order to compare and tally the amount of novel information observed in each subsequent interview. Saturation was achieved when no novel information was gathered. Concepts or concerns that emerged were used to determine revisions made to the LFA-REAL PRO as described above. All revisions—and the rationale behind the revisions—were tracked using an item-tracking matrix.

## Results

### Quantitative results

#### Recruitment

Of the 665 respondents to the online screening questions, 435 completed the online screening, 216 were found to be eligible, 30 were enrolled in the study, and 25 completed their interview.

#### Demographics of the study sample

Self-reported sociodemographic characteristics were collected on the “Questions about You” form at the completion of the interview. The results are presented in Table 1.

**Table 1:** Participant Socio-demographic Characteristics

Participant Characteristics	Total *N* = 25	Phase 1 *N* = 10	Phase II *N* = 15
Age (years)			
Mean (SD)	45.7(13.5)	42.6 (13.8)	47.7(13.5)
Gender, n (%)			
Female	22 (88.0%)	8 (80.0%)	14 (93.3%)
Ethnicity, n (%)			
Hispanic	5 (20.0%)	3 (30.0%)	2 (13.3%)
Race, n (%)^1^			
American Indian or Alaska Native	3 (12.0%)	1 (10.0%)	2 (13.3%)
Asian	1 (4.0%)	–	1 (6.7%)
Black or African American	7 (28.0%)	3 (30.0%)	4 (26.7%)
Native Hawaiian or other Pacific Islander	2 (8.0%)	–	2 (13.3%)
White	11 (44.0%)	6 (60.0%)	5 (33.3%)
Other^2^	5 (20.0%)	1 (10.0%)	4 (26.7%)
Marital status, n (%)			
Single	7 (28.0%)	2 (20.0%)	5 (33.3%)
Married	11 (44.0%)	6 (60.0%)	5 (33.3%)
Divorced	4 (16.0%)	–	4 (26.7%)
Separated	1 (4.0%)	1 (10.0%)	–
Widowed	1 (4.0%)	–	1 (6.7%)
Other^3^	1 (4.0%)	1 (10.0%)	–
Current living/domestic situation, n (%)			
Living alone	4 (16.0%)	1 (10.0%)	3 (20.0%)
Living with a spouse, partner, family, or friends	20 (80.0%)	9 (90.0%)	11 (73.3%)
Other^4^	1 (4.0%)	–	1 (6.7%)
Employment status, n (%)^1^			
Employed, full-time	6 (24.0%)	3 (30.0%)	3 (20.0%)
Employed, part-time	5 (20.0%)	2 (20.0%)	3 (20.0%)
Homemaker	2 (8.0%)	1 (10.0%)	1 (6.7%)
Student	1 (4.0%)	–	1 (6.7%)
Unemployed	2 (8.0%)	–	2 (13.3%)
Retired	2 (8.0%)	–	2 (13.3%)
Disabled	12 (48.0%)	6 (60.0%)	6 (40.0%)
Highest level of education, n (%)			
Secondary/high school	2 (8.0%)	1 (10.0%)	1 (6.7%)
Technical or vocational degree	2 (8.0%)	–	2 (13.3%)
Some college/university	7 (28.0%)	5 (50.0%)	2 (13.3%)
College/university degree (BA, BS)	7 (28.0%)	3 (30.0%)	4 (26.7%)
Postgraduate degree (MA, PhD)	6 (24.0%)	1 (10.0%)	5 (33.3%)
Other^5^	1 (4.0%)	–	1 (6.7%)
General health status within the past week, n (%)			
Excellent	–	–	–
Very Good	1 (4.0%)	1 (10.0%)	–
Good	12 (48.0%)	5 (50.0%)	7 (46.7%)
Fair	10 (40.0%)	4 (40.0%)	6 (40.0%)
Poor	2 (8.0%)	–	2 (13.3%)

The mean age of participants was 45.7 years (± 13.5), the majority were female (*n* = 22; 88%) and 44% (*n* = 11) identified themselves as “White”. Eleven participants were married (44%), seven were single (28%), and four were divorced (16%). Twenty participants (80%) were living with a spouse, partner, family, or friends, while four (16%) lived alone. When asked about employment status, patients were disabled (*n* = 12; 48%), employed full-time (*n* = 6; 24%), or employed part-time (*n* = 5; 20%). Seven had some college or university experience (28%), seven had a college or university degree (28%), and six had obtained a postgraduate degree (24%).

Participants described their health within the past week as good (*n* = 12; 48%) or fair (*n* = 10; 40%). The mean length of time since diagnosis that patients reported was 16.4 years (SD 11.9) ago, with a range of 1 to 47 years. The most commonly reported other conditions include the following: depression (n = 12; 48%), fibromyalgia (*n* = 11; 44%), and anxiety (*n* = 10; 40%).

#### Self-reported clinical characteristics of the study sample

Self-reported clinical information is included in Table 2.

**Table 2:** Participant Clinical Characteristics ^1^

Participant Characteristics	Total *N* = 25*	Phase 1 *N* = 10	Phase II *N* = 15
Doctor(s) seen for lupus^1^			
Primary care or general doctor	16 (64.0%)	7 (70.0%)	9 (60.0%)
Lupus specialist or rheumatologist	25 (100%)	10 (100%)	15 (100%)
Skin doctor or dermatologist	10 (40.0%)	4 (40.0%)	6 (40.0%)
Kidney doctor or nephrologist	13 (52.0%)	6 (60.0%)	7 (46.7%)
Treatments or medications taken for lupus^2^			
Hydroxychloroquine (Plaquenil), Quinacrine (Atabrine), or Chloroquine	24 (96.0%)	10 (100%)	14 (93.3%)
Azathioprine (Imuran)	3 (12.0%)	3 (30.0%)	0 (0%)
Mycophenolate Mofetil (CellCept), or Mycophenolic acid or MMF	17 (68.0%)	9 (90.0%)	8 (53.3%)
Tacrolimus, Sirolimus or Cyclosporin	4 (16.0%)	2 (20.0%)	2 (13.3%)
Cyclophosphamide (Cytoxan)	10 (40.0%)	5 (50.0%)	5 (33.3%)
Belimumab (Benlysta)	8 (32.0%)	4 (40.0%)	4 (26.7%)
Methotrexate	9 (36.0%)	2 (20.0%)	7 (46.7%)
Prednisone or other steroids	23 (92.0%)	10 (100%)	13 (86.7%)
Other^3^	8 (32.0%)	1 (10.0%)	7 (46.7%)
Lupus has previously or currently affected kidneys			
Yes	15 (60.0%)	7 (70.0%)	8 (53.3%)
No	7 (28.0%)	2 (20.0%)	5 (33.3%)
Don’t know	3 (12.0%)	1 (10.0%)	2 (13.3%)
Currently on dialysis			
Yes	3 (12.0%)	2 (20%)	1 (16.7%)
No	22 (88.0%)	8 (80.0%)	14 (93.3%)
Experienced being hospitalized for more than 24 h because of a lupus flare			
Yes	19 (76.0%)	9 (90.0%)	10 (66.7%)
No	6 (24.0%)	1 (10.0%)	5 (33.3%)

Participants indicated that they consulted a variety of physician specialties for lupus: 100% were seeing a lupus specialist or rheumatologist, 52% a nephrologist (*n* = 13), and 40% a dermatologist (*n* = 10). Participants also reported a variety of different treatments or medications including antimalarials (*n* = 24; 96%); prednisone or other steroids (*n* = 23; 92%); and mycophenolate mofetil (CellCept or MMF) or mycophenolic acid (*n* = 17; 68%). Fifteen participants (60%) reported that lupus previously or currently affected their kidneys. Nineteen participants (76%) reported a history of being hospitalized for more than 24 h because of a lupus flare.

#### Clinical diagnosis

Nineteen participants (76%) had their lupus diagnosis confirmed by a subsequent contact with their treating physician, including seven participants in Phase I and twelve participants in Phase II. Based on their treating physicians’ report, the average time since diagnosis was 13.4 years (SD 10.8) with a range of 1–39 years. For participants without a clinician confirmed diagnosis (the treating physician could not be reached, *n* = 6), all reported being seen by a rheumatologist and being currently treated with antimalarials and/or mycophenolate mofetil.

#### SF-36 domains: frequency distribution

The results of the SF-36 Version 1.0 Standard RAND scoring, ranged from 0 (worst possible level of functioning) to 100 (best possible level of functioning).

The highest mean scores were in the domain of emotional well-being, and the lowest mean score was for role limitations due to physical health. The total sample’s overall mean score for the eight different domains of the SF-36 were as follows: emotional well-being 68.8 (SD 20.2); role limitations due to emotional problems 62.7 (SD 41.2); social functioning 54.5 (SD 31.4); physical functioning 49.0 (SD 25.8); pain 41.2 (SD 24.4); general health 35.6 (SD 23.2); energy or fatigue 31.0 (SD 15.9); and role limitations due to physical health 24.0 (SD 34.2).

Scores were lower in the physical component summary (PCS; 29.8 [SD 9.1]) than in the mental component summary (MCS; 46.4 [SD 11.6]).

#### Qualitative results – phase I (concept elicitation)

Clinicians’ reports and the LFA preliminary work that was further probed in Phase I showed that patients spontaneously report joint pain, joint swelling and joint stiffness. By explaining inflammatory arthritis to patients as a combination of all these joint symptoms, we may increase the chances that patients will actually describe inflammatory, lupus related, arthritis.

It seems that at least 5/8 patients with arthritis, 7/10 patients with fatigue, and 5/8 patients with rash were able to attribute their symptoms to SLE.

#### Symptom experience

Concepts (symptoms), reported by the participants are presented in Table 3 and show saturation of symptoms in the 10 concept elicitation interviews. After the completion of the first interview, no new symptoms were reported. The most common symptoms were joint or bone pain, stiffness, or swelling, and fatigue. Fatigue was reported by all participants (*n* = 10, 100%), 50% spontaneously and 50% elicited. The following additional symptoms were reported by greater than or equal to 50% of the sample: swelling (*n* = 9), weakness (*n* = 9), difficulty breathing/shortness of breath (*n* = 8), skin problems (*n* = 8), chest pain (*n* = 7), tiredness (*n* = 6), hair loss (*n* = 6), muscle pain, aching, or stiffness (*n* = 5), and fever (*n* = 5). For each symptom, the nature of symptoms (i.e., constant versus variable) and the likelihood of being able to clearly attribute the symptoms to SLE was probed. Patients believed they were able to ascertain that symptoms were due to lupus. The likelihood that they were correct was substantiated by their ability to report the waxing/waning nature of these symptoms.

**Table 3:** Saturation grid

SYMPTOM SATURATION s = spontaneous p = probed u = don t know							Participants									
	Male (M)/ Female (F)						F	F	M	F	F	F	F	F	F	M
	Race / Ethnicity						Hispanic/Latino	Black/Afr ican Am	White	Black/Afr ican Am	Hispanic/Latino	White	Am Indian/AIaskan Nat	Black/Afr ican Am	Asian/Other	Hispanic/Latino
	Interview Date						18-Mar	23-Mar	23-Mar	24-Mar	24-Mar	25-Mar	29-Mar	29-Mar	4-Apr	5-Apr
	IDs (*N* = 8)						201–001	201–004	201–006	101–001	201–005	201–003	101–003	101–002	201–008	201–007
	n	%	n	%	n	%										
	OVERALL		SPONTANEOUS		PROBED											
Joint/bone pain, stiffness or swelling (arthritis, arthritislike symptoms)	10	100%	10	100%	0	0%	S	s	s	s	s	s	s	s	s	s
Fatigue	10	100%	5	50%	5	50%	S	s	P	P	P	P	s	s	s	P
Swelling	9	90%	6	60%	3	30%	P	p	s	s	s	s	p	s	s	
Weakness	9	90%	2	20%	7	70%	P	p	s	p	p	p	p		s	P
Difficulty breathing/shortness of breath	8	80%	5	50%	3	30%	P		s	p	s	s	s		p	S
Skin problems (e.g., rash, pigmentation)	8	80%	5	50%	3	30%	s		u	s	s	s	p	s	p	P
Chest pain	7	70%	2	20%	5	50%	p			p	p	s	u	s	p	P
Tiredness	6	60%	4	40%	2	20%	s		p		s	s		u	p	s
Hair loss	6	60%	2	20%	4	40%	p	p	p	s			u	s	p	u
Muscle pain, aching or stiffness	5	50%	2	20%	3	30%	p			p	s	p	s	u		
Fever	5	50%	2	20%	3	30%	s	s		p		p				p
Other*	7	70%	7	70%	0	0%	s	s	s	s	s	s	s		s	s

#### Qualitative results – phase II (cognitive debriefing interviews)

This section presents the findings from the 15 cognitive debriefing (CD) interviews conducted in order to assess the content validity of the LFA-REAL PRO.

The qualitative interviews were semi-structured in that they were guided by a framework of themes dictated by the draft instrument while also allowing for new ideas. Every attempt was made to ask the same questions to each participant; however, given that the interview was not rigidly scripted and that some participants spent more time elaborating on certain themes than others, participants did not necessarily provide responses to all of the same questions. Therefore, the denominator varied across questions and has been included for each section to reflect the number of participants that responded to each question.

The majority of instructions and questions in the instrument were found to be clear and concise and could be paraphrased correctly by most patients. Almost all patients (14/15) reported that the questionnaire was easy to use, and that the order of the items was easy to follow. However only 60% marked the forms correctly, leading to adjustments in the instructions to help increase clarity. All patients thought all the items on the PRO were relevant to patients with lupus. The draft PRO used a 3-point (0–3), 100 mm visual analog scale (VAS); with 0 being “None” and 3 being “Worst imaginable”, and landmarks at 1 and 2 where 0–1 is mild, 1–2 moderate, and 2–3 severe. All patients asked were able to correctly paraphrase “None,” “Mild,” “Moderate,” “Severe” and “Worst imaginable.” All patients asked were also able to find an appropriate response to the questions and thought the response options were appropriate. Although 6 patients suggested changing the response options to a 0–10 scale, the majority of patients found the 0–3 response scale appropriate for segmenting mild, moderate and severe disease activity. Therefore, the 0–3 point VAS was retained. Lastly, patients reported fluctuations in symptoms but responded well to the one-month recall period. A summary of the CD interviews is presented in Table 4. The main changes made to the PRO were clarifications to the instructions. The Flesch reading ease score for the PRO is 71.5 (in the recommended range of 60–80) and the Flesch-Kincaid grade level is 7.

**Table 4:** Cognitive Debriefing

Area probed	N (%) Understood	Comments
Instructions		
Section 1 “the past month”	14/15 (93)	
Section 2 “current lupus symptoms”	9/15 (60)	Some difficulty with “other health problems”
Section 3 “if you filled this form before”	15/15 (100)	
Examples	11/15 (73)	“Examples were helpful”
Recall Period −1 month	13/15 (87)	Thought it was too long (n = 2)
Response options		
Scale	14/15 (93)	Easy to use
Word anchors and numbers	15/15 (100)	No difficulty
Meaning of the word anchors	5/5 (100)	Understood the meaning
Symptoms		
Rash	15/15 (100)	Understood the meaning
Arthritis-joint pain	14/14 (100)	
Arthritis - joint stiffness	15/15 (100)	
Arthritis-joint swelling	15/15 (100)	
Arthritis-overall	14/15 (93)	Understood the meaning
Muscle pain oraches	14/15 (93)	
Body Symptoms	Difficulty	The wording was changed
Chest pain	13/15 (87)	
Shortness of breath	13/15 (87)	
Swelling in legs	12/15 (80)	
Other	No issues	“Not sure how to factor in all symptoms”
Fatigue	15/15 (100)	
Fever	15/15 (100)	
Hair loss	15/15 (100)	
Use of the scale Verbalized understanding of the use of the scale	15/15 (100)	Correctly used the scale (n = 9), circled number instead of a line to mark response (n = 4), incorrect use of response option (n = 2)

The revised PRO presented in Fig. 1 contains 11 items that investigate rashes, arthritis (joint pain/stiffness/swelling), muscle pain, symptoms from inside the body, fatigue, and hair loss.

## Discussion

This paper describes the methods and results of qualitative research with lupus patients used to ensure content validity of the LFA REAL™ PRO, and supports our hypothesis that a patient-centered approach can be used to evaluate the content validity of the LFA-REAL PRO. The final draft of this instrument is shown in Fig. 1 as a series of simple, landmarked VAS global assessments of symptoms that are confirmed as being commonly experienced and important to SLE patients, in terms that they understand and approve of. Instructions are brief and guide the patient to a consideration of what represents active lupus disease as opposed to longstanding symptoms that are more likely due to damage. A global score and organ-based or symptom-specific evaluations can be obtained from the patient’s perspective that is comparable to the physician’s ratings of the most common lupus features on the partnered CLINRO [29, 30].

Routine use of PROs will enhance clinical practice. Unfortunately, in lupus, PROs in use have been problematic to execute and interpret. In trials they often provide disparate outcomes. We hope that a measurement that complements the physician’s judgment will be able to increase the use of PROs in routine clinical care.

The poor correlation between PROs and physician assessments of disease activity or disease severity tell us that PROs are measuring information not captured in physician assessments. PROs measure the impact of disease on patients’ daily lives. Disease activity as assessed by physicians is based on symptoms, signs and laboratory assessments that are proximal to the impact on daily lives. Current PROs used in SLE emphasize these impacts rather than symptoms and disease activity. The Lupus Impact Tracker (LIT) [31, 32] derived from the LupusPRO [33, 34] was shown to be responsive to physician assessed changes in disease activity (SLEDAI, PGA, and SRI), but also to patient assessed changes in healthy (HRQoL), and disease activity (SLAQ). LIT has also been shown to be responsive to physician assessed treat to target health outcomes being used recently in SLE [35]. As expected, achieving lupus low disease activity and remission correlates with improvements in QoL. Although, PROs – including the LIT- have not shown any association with damage measures [36] it is possible that lupus PROs are contaminated by damage when reports of pain refer to pain from joints affected by avascular necrosis or osteoarthritis. The LFA-REAL PRO was designed to mainly evaluate disease activity related physical health and not impacts on daily life or SLE related damage. While the LFA-REAL does not cover the full spectrum of disease activity in the listed items, the “is there anything else to report” can include any additional issues deemed important by the patient and can inform further changes to the instrument. Mental health, impacts and damage are clearly important and should continue to be measured and addressed in lupus care and research.

A ClinRO disease activity instrument, the Lupus Activity Index (LAI), was developed as a physician measure that incorporates, along with a number of other components, eight visual analog scales which are restricted to 4 symptoms (fatigue, rash, joint involvement, serositis) and 4 signs (neurologic, renal, pulmonary, and hematologic involvement) [31, 32]. The LFA-REAL was designed to include both the physician and patient perspectives of disease activity and allow for a simple yet comprehensive evaluation of any and all active features, individually and in composite. Unlike the LAI, which uses the landmarks as flags to suggest approximate grades of disease severity the LFA-REAL uses these landmarks as defined cut-offs (mild disease is < 1) [29, 30]. This promotes more consistency in scoring between observers and gives the same line length to adjust gradations of mild, moderate or severe manifestations. The ClinRO pre-specifies the most commonly affected organ/systems/symptoms of lupus but leaves room to expand the list for individual patients. The PRO described here has been refined with patient input to include the symptoms that are important to patients but overlaps with the ClinRO in specified ways to elicit an assessment that might correlate to what the physicians evaluate.

We readily acknowledge several limitations, the SLE diagnosis was not confirmed for all participants, the PRO may not cover all aspects of SLE, data on fibromyalgia was not collected. During the development of the draft PRO, which was tested in the current study, volunteer patients had listed disease features that were most important to them, and it became clear that, unlike rash, the concept of arthritis is not universally understood. However, patients do have a granular understanding of each feature that a physician incorporates into an assessment of inflammatory arthritis, such as joint pain, swelling, and stiffness. For this reason, the patients are instructed to rate these components separately and then, only after that, to factor them together in a global arthritis rating. We hypothesize that this may produce an understanding of arthritis more equivalent to that of a trained clinician than previous PROs have been able to achieve. If so, it might be easier to determine whether discrepancies between physician and patients’ perception of improvement are due to different interpretations of the question or different assessments of the same domain. For example, if swelling is markedly improved, but pain is not, this will be apparent in the arthritis section of the PRO where it might not be as apparent to physicians when completing the CLINRO.

To complete the PRO in a consecutive series of visits, patients are instructed to begin a new scoring session with review of what they scored at the last visit and to consider whether they would rate each scale as same, improving, or worsening by moving their mark (or not) along the line accordingly. This approach has been shown to improve the consistency of scoring PGAs in lupus studies and is mandated for use in the SELENA SLEDAI PGA that clinicians routinely score [37, 38]. By providing an equivalent scoring methodology for clinicians and patients for the most common lupus manifestations, the LFA-REAL may offer additional insights into the physician-patient discordance that has so often been observed in trials [39].

In the next steps, assessment of the quantitative performance of LFA-REAL PRO scores will include tests of reliability, validity, and responsiveness, including correlations of the REAL PRO scores with those of other PRO measures used in lupus clinical trials (e.g. SF36, Lupus QOL, PtGA).

## Conclusion

SLE is an unpredictable condition with a variable course and prognosis ranging from mild, manageable disease to a severe, life threatening disorder. Some patients may experience predominantly skin manifestations, while others can have musculoskeletal involvement, renal disease, neurological involvement, pericarditis, pleurisy or solid organ inflammation, or a combination of these [40]. A chronic, waxing and waning, complicated disease such as lupus requires a tool that is not only responsive and dimensional but also resonant to the concerns of the lupus population without the burden of many layers of complexity. By directing the patient’s focus to active disease symptoms, the LFA-REAL PRO may help make the assessment of treatments both in trials and clinical practice more efficient, accurate, and useful. Furthermore, the PRO may provide important information about the direction of progress for the whole patient, as well as individual features of disease. Given the known negative correlation of disease activity with quality of life [41], this may add to the understanding of which features have the greatest impact on a person’s experience.

## Additional file

Additional file 1: Participant Flowchart. (PDF 57 kb)

## Abbreviations

BILAG: British Isles Lupus Activity Group
CRFs: Case report forms
LFA: Lupus Foundation of America
LFA-REAL™: Lupus Foundation of America Rapid Evaluation of Activity in Lupus
OCR: Optical character recognition
PGA: Physician’s Global Assessment
PRO: Patient-reported outcome
SLEDAI: SLE Disease Activity Index
VAS: Visual analog scales
CD: Cognitive debriefing
LIT: Lupus Impact Tracker
SRI: SLE responder index
SLAQ: Systemic Lupus Activity Questionnaire
HRQoL: Health-related quality of life
BILD: Brief index of lupus damage
LAI: Lupus Activity Index
