# Supplementary figures and images for: Development and content validity of the Lupus Foundation of America rapid evaluation of activity in lupus (LFA-REAL™): a patient-reported outcome measure for lupus disease activity
Source: Health Qual Life Outcomes. 2019 Jun 7;17:99. doi: 10.1186/s12955-019-1151-8 (PMC6555910; doi:10.1186/s12955-019-1151-8)

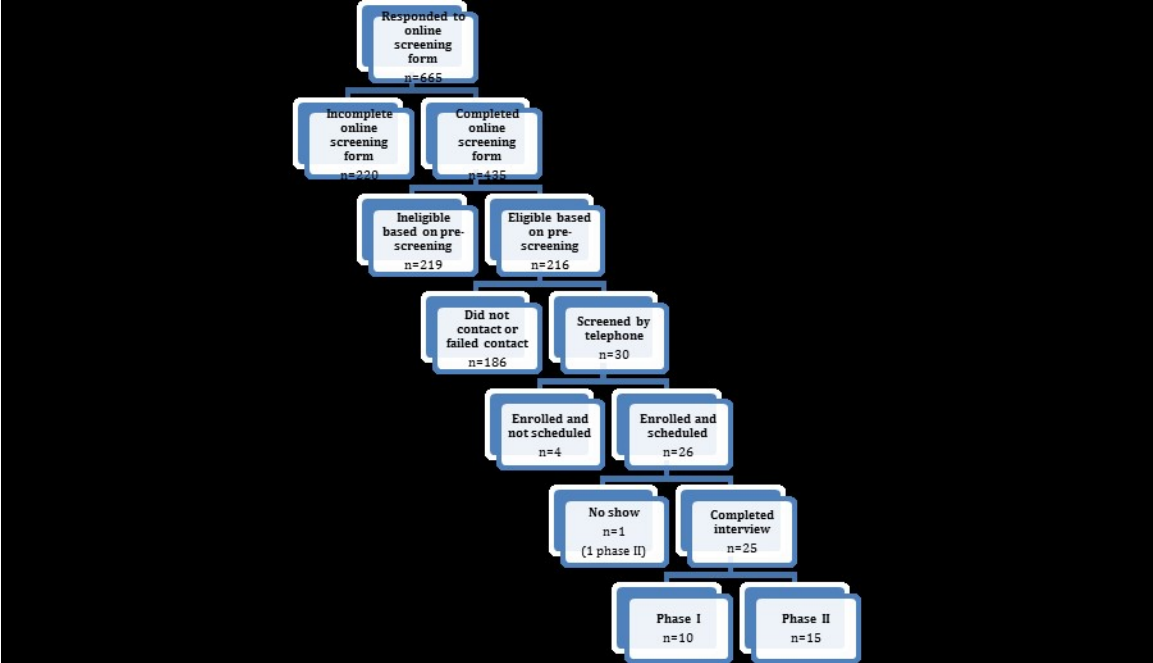

Supplement: Supplementary file 1 — Participant Flowchart. (PDF 57 kb) [file 12955_2019_1151_MOESM1_ESM.pdf]
